# A Brief Participatory Workplace Intervention on Dietary Barriers and Healthy Eating Intentions Among Employees: A Pilot Study

**DOI:** 10.3390/nu17213371

**Published:** 2025-10-27

**Authors:** Aleksandra Hyży, Ilona Cieślak, Joanna Gotlib-Małkowska, Mariusz Panczyk, Mariusz Jaworski

**Affiliations:** Department of Education and Research in Health Sciences, Medical University of Warsaw, 02-091 Warszawa, Poland; aleksandra.hyzy@wum.edu.pl (A.H.); joanna.gotlib@wum.edu.pl (J.G.-M.); mariusz.panczyk@wum.edu.pl (M.P.);

**Keywords:** diet, workplace intervention, healthy eating promotion, educational intervention, behavioral intervention

## Abstract

Background: An unhealthy diet is a major modifiable risk factor for chronic disease, and workplace environments may pose specific barriers to healthy eating. Methods: This single-arm, pre–post workplace intervention assessed short-term changes in perceived dietary barriers (primary outcome) and healthy eating intentions (secondary outcome) among school employees (*n* = 36; 83.3% women; mean age = 46.8 years). The 60 min participatory session integrated behavioral economics principles, practical nutrition exercises, and psychological strategies designed to enhance self-efficacy, optimism, and resilience. Results: The total perceived barrier score decreased significantly (Δ = −1 [IQR −2 to 0]; the paired Wilcoxon signed-rank test = −4.689, *p* < 0.001, *r* = −0.78). Post-intervention (T2), the proportion of participants reporting an intention to prepare healthy meals increased (McNemar’s test, *p* < 0.001; OR = 10.0, 95% CI 1.8–54.5). The session was rated as highly useful (mean = 8.3/10), and at the three-week follow-up (T3), 91.7% of participants reported maintaining at least one dietary change. Conclusions: Although limited by its single-arm design and short follow-up, this pilot study suggests that a brief, participatory behavioral intervention may be a feasible and acceptable approach to support healthier eating in the workplace.

## 1. Introduction

### 1.1. Background and Public Health Context

An unhealthy diet is a major risk factor for noncommunicable chronic diseases, including obesity, type 2 diabetes, and cardiovascular, all of which pose global health and economic challenges [1]. According to the World Health Organization (WHO), noncommunicable diseases account for 74% of all deaths worldwide, and inadequate nutrition is one of the key modifiable risk factors [2]. The Organization for Economic Cooperation and Development (OECD) estimates that unhealthy lifestyles, including poor dietary habits, cost healthcare systems in its member countries an average of 3.3% of gross domestic product (GDP) annually and contribute to productivity losses due to absenteeism and presenteeism [3].

### 1.2. Workplace as a Setting for Dietary Interventions

Healthy nutrition in the workplace can contribute to improving employees’ overall health, enhancing their productivity, and reducing costs for employers and public healthcare systems [4]. Despite growing awareness of the importance of a healthy lifestyle and various interventions promoting healthy eating at work [5], many employees still struggle to adhere to dietary recommendations in this setting [6,7,8].

Previous studies have identified multiple barriers that hinder the adoption of healthy eating habits at work. Observational studies indicate that the most frequently reported challenges include a lack of time to consume or prepare meals, work-related stress, limited access to healthy food options, and irregular eating patterns, including intentional meal skipping [7,8].

### 1.3. Barriers to Healthy Eating in Occupational Settings

The perception of preparing healthy meals as time-consuming and organizationally demanding has been identified as a major barrier, potentially discouraging individuals from engaging in health-promoting behaviors, even among those who report positive intentions to change [7]. Comparative analyses of randomized intervention trials support this idea, demonstrating that many workplace dietary choices are made quickly and semi-consciously, with strong environmental influences, such as product presentation or availability [4,6]. Furthermore, a narrative review by Rosin et al. [9] reveals that these barriers are rooted in the organizational structure of workplaces and insufficient infrastructure promoting healthy choices.

### 1.4. Teachers and School Staff as a Priority Group

These barriers are general in nature and observed across various occupational sectors. However, certain groups of employees appear particularly susceptible. Among them are school personnel, including teachers and administrative staff, whose daily involves high cognitive demands, limited break times, and insufficient infrastructure to support healthy dietary choices. Research increasingly emphasizes that schools, beyond their pivotal role in promoting student health, also represents an important environment for influencing adults [10]. However, interventions targeting school employees directly have been studied only to a limited extent. A systematic review of over 12,000 records and six unique studies found that interventions addressing diet, physical activity, and sleep among teachers and other educational staff were largely ineffective, yielding mixed and often statistically insignificant results [10]. This underscores the need for further research in this occupational group, which is underrepresented in the literature.

### 1.5. Key Determinants of an Effective Workplace Nutrition Intervention

In light of these challenges and limitations, it is especially important to search for novel intervention approaches that account for the actual mechanisms underlying dietary decision-making, particularly those derived from behavioral economics. Unlike classical economic theories, which assume individuals make fully rational choices, behavioral economics considers factors such as the status quo effect (the tendency to maintain existing choices), framing (how information is presented to shape perception), decision fatigue (the decline in decision quality as the number of decisions increases), and limited willpower (finite self-control resources) [11]. Based on these principles, researchers have developed the concept of choice architecture. This concept refers to designing decision-making environments in ways that facilitate healthier choices without imposing them.

In the context of workplace nutrition, behavioral economics is a particularly relevant framework because employees often make food choices when working automatically, and out of convenience rather than deliberation due to time constraints. This approach introduces subtle cues and environmental prompts (nudges) to help align habitual decisions with long-term health goals, eliminating the need for constant self-control or organizational restructuring. Moreover, behavioral economics-based strategies are low-cost, scalable, and easily integrated into existing workplace settings, making them suitable for feasibility-oriented interventions targeting school employees.

Examples of such applications include traffic light labeling, placing healthier options at eye level or near checkout points, and reducing the visibility of less favorable options. These interventions have proven effective in workplace cafeterias and institutional food environments [4,12].

Although choice architecture mechanisms can mitigate the impact of environmental barriers, they cannot eliminate them entirely. The effectiveness of this approach can be substantially enhanced by tailoring interventions to participant profiles and actual needs [6,13]. Therefore, a diagnostic component that identifies both declarative and psychological obstacles (e.g., ambivalence) should precede the development of intervention content. This diagnosis allows for the design of interventions that genuinely address the challenges and readiness for change within a given group.

In this context, to the approach to promoting healthy eating described by Contento [13] as needs-based, learner-centered, and behaviorally focused (NLB approach) is of particular importance. This framework assumes that effective nutrition interventions should be developed in stages with the active participation of the target group. They should begin with the identification of individual barriers, resources, and goals. The NLB approach emphasizes the importance of content personalization and co-creating interventions with those directly affected. Such collaboration fosters greater engagement, a sense of agency, and intrinsic motivation, aligning tools more effectively with everyday realities. Moreover, it reduces resistance to change and supports the implementation of behavioral strategies.

In a personalized approach to promoting healthy eating in the workplace, one of the key elements is to consider the psychological resources of the participants, as they may significantly influence the effectiveness of the actions undertaken. Research shows that these factors affect readiness for and ability to sustain change in the face of environmental barriers. The most frequently examined psychological resources are optimism, self-efficacy, and resilience.

According to Bandura’s theory, self-efficacy refers to an individual’s belief in their ability to act effectively in specific situations [14]. Lombardo et al. [15] demonstrated that individuals with higher self-efficacy and stronger emotional regulation skills were more likely to engage in health-promoting behaviors. These findings suggest that the capacity for emotional reappraisal and suppression may encourage healthier choices, especially among overweight or obese individuals.

Dispositional optimism, as described by Carver and Scheier [16], is associated with the expectation of positive outcomes and perseverance in action. Ait-Hadad et al. found that higher levels of dispositional optimism were linked to a better diet quality and more frequent consumption of healthy foods (e.g., vegetables, whole grains, fish) [17].

According to Luthar et al., resilience refers to the adaptive capacity in the face of stress and adversity [18]. Sinska et al. demonstrated that resilience is positively associated with adaptive dietary patterns and a reduced tendency toward emotional eating under pandemic-related stress [19]. Similar findings were reported by Sampson et al., who demonstrated that higher levels of resilience and resilient coping were associated with better dietary quality, increased physical activity, and adequate sleep duration, especially under conditions of chronic stress [20].

Despite the growing number of studies on the effectiveness of educational and environmental health interventions, existing workplace nutrition promotion approaches have primarily focused on specific factors that influence dietary decisions. These factors include psychological determinants, behavioral economic mechanisms, and tailoring content to participants’ needs. However, research integrating these perspectives is lacking, particularly in occupational settings with high cognitive demands, limited break times, and specific organizational challenges, such as schools [10]. However, studies combining behavioral economics principles with individual psychological resources and educational approaches grounded in needs assessment and co-creation of intervention content (the NLB approach) remain scarce. Our study aimed to address this gap by designing a workplace intervention to promote healthy eating.

### 1.6. Aim of the Study

The aim of this study was to assess the short-term effects of a brief, participatory workplace intervention designed to promote healthy eating by reducing dietary barriers and strengthening the intentions to engage in health-promoting behaviors.

## 2. Theoretical Framework and Study Hypotheses

**Figure 1:**
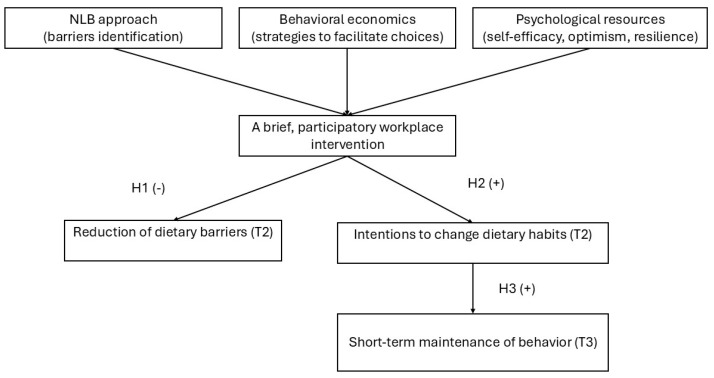
Theoretical framework of the Brief Participatory Behavioral and Educational Workplace Intervention (BPBEWI) integrating the NLB approach, behavioral economics principles, and psychological resource enhancement (self-efficacy, optimism, and resilience). Abbreviations: NLB—needs-based, learner-centered, and behaviorally focused approach.

The study’s theoretical framework integrated three complementary components that collectively supported the intervention’s overarching goal of promoting health-enhancing dietary behaviors in the workplace (Figure 1).

First, the NLB approach [13] was applied to identify barriers to healthy eating in the workplace and design tailored actions for participants. This approach ensured that the intervention addressed real-life challenges perceived by employees, thus enhancing the relevance and the motivation to change.

Second, the intervention incorporated behavioral economics principles, such as simplification, default options, and decision guidance. These principles facilitate healthier food choices under the cognitive and time constraints of real life. These mechanisms aligned with the COM-B (Capability, Opportunity, Motivation → Behavior) model because as they increased participants’ psychological capability and reflective motivation to engage in healthy dietary practices.

Third, the intervention included elements aimed at strengthening psychological resources such as self-efficacy, optimism, and resilience [14,15,16,17,18,19,20], which, according to Self-Determination Theory (SDT), enhance intrinsic motivation and sustained behavioral engagement [21]. The intervention was designed to support self-regulated behavior change by fostering competence and autonomy.

Researchers operationalized these components through specific Behavior Change Techniques (BCTs), including problem solving (BCT 1.2), action planning (BCT 1.4), and verbal persuasion about capability (BCT 15.1) [22]. The cognitive and participatory activities aimed to reduce perceived barriers (−), increase perceived knowledge (+), and strengthen intentions to adopt healthier eating practices (+).

Based on this theoretical integration, the research team formulated the following hypotheses:

**H1.**  *Participation in the intervention will reduce perceived dietary barriers (−)*.

**H2.**  *Participation in the intervention will increase perceived nutrition-related knowledge and intentions to improve eating habits (T2) (+)*.

**H3.**  *Positive short-term post-intervention changes (T2) will be maintained at the three-week follow-up assessment (T3) (+)*.

Figure 1 shows the overall logic model, which illustrates how the NLB, behavioral economics, and psychological resources interact to produce changes in the mediating mechanisms (knowledge, self-efficacy, and motivation) that lead to improvements in dietary intentions and perceived barriers.

## 3. Materials and Methods

### 3.1. Study Type

This study employed a quasi-experimental design, specifically a one-group pre-test–post-test design [23]. The study included three measurement points: baseline (T1), post-intervention (T2), and a three-week follow-up (T3). Recruitment took place from 7 April 2025 to 18 April 2025. The pre-intervention assessment took place on 21 April 2025. The post-intervention assessment took place on 28 April 2025, and the three-week follow-up was completed on 19 May 2025. No deviations from the study protocol were recorded during the intervention.

The manuscript was prepared in accordance with the TREND (Transparent Reporting of Evaluations with Nonrandomized Designs) statement [24] and the TIDieR (Template for Intervention Description and Replication) checklist, incorporating [25] all required elements into the study description.

### 3.2. Participants and Recruitment Process

The school selection for this study was conducted in Warsaw, Poland, which is divided into 18 administrative districts. One district was randomly selected (the name of the district has been blinded for reporting purposes). Within that district, the research team identified and invited eight public primary schools to participate in this study. Two schools agreed to participate, yielding an acceptance rate of 25%. The research team implemented the random selection of the district and the standardized school recruitment procedure to minimize selection bias while maintaining the feasibility of conducting this study in a real educational setting.

Eligibility criteria for participation included the following: (a) employment at one of the two educational institutions involved in this study, (b) engagement in white-collar work (e.g., teachers, administrative specialists, managerial staff), (c) presence in the workplace on the day of the intervention, and (d) provision of voluntary informed consent to participate. Exclusion criteria were (a) employment limited to manual or auxiliary tasks (e.g., technical or cleaning staff), (b) absence from work on the day of the intervention (e.g., due to vacation or sick leave), and (c) refusal to provide consent to participate.

The research team conducted participant recruitment internally within the schools. All eligible individuals meeting were informed about this study during staff meetings and via email from the school administration. The final composition of the groups was determined based on lists of individuals who expressed a willingness to participate. These lists were provided by the school principals to the intervention facilitator. The principals had previously been informed about the inclusion and exclusion criteria.

Restricting the sample to white-collar employees ensured greater homogeneity of the study group with regard to cognitive demands, work patterns, and dietary behaviors. White-collar employees often have similar workplace eating habits (e.g., meal consumption) and are exposed to specific environmental stressors. They may require different educational strategies than individuals engaged in manual labor.

Participation was voluntary and not linked to employee evaluations or material benefits. Any personal data or information disclosed during the practical component (e.g., regarding health barriers or psychological resources) was treated as confidential. Employers had no access to individual questionnaire results or data on participants’ psychological characteristics. Data were analyzed and reported exclusively in aggregated form.

Of the 68 eligible employees invited to participate, 52 provided written informed consent and completed the baseline assessment (participation rate: 76.5%). At post-intervention assessment (T2), 45 participants submitted questionnaires, and 36 completed all three assessment points, resulting in a final retention rate of 69.2%. No withdrawals were related to dissatisfaction with the intervention.

To evaluate potential selection bias, we compared the baseline characteristics of the completers (*n* = 36) and the non-completers (*n* = 16). There were no significant differences between the groups in terms of age (*p* = 0.47), gender distribution (*p* = 0.64), educational level (all participants had a degree of higher education), or the baseline number of perceived dietary barriers (*p* = 0.53). These results suggest that attrition was primarily related to logistical reasons rather than systematic differences between participants, which minimizes the likelihood of selection bias.

**Figure 2:**
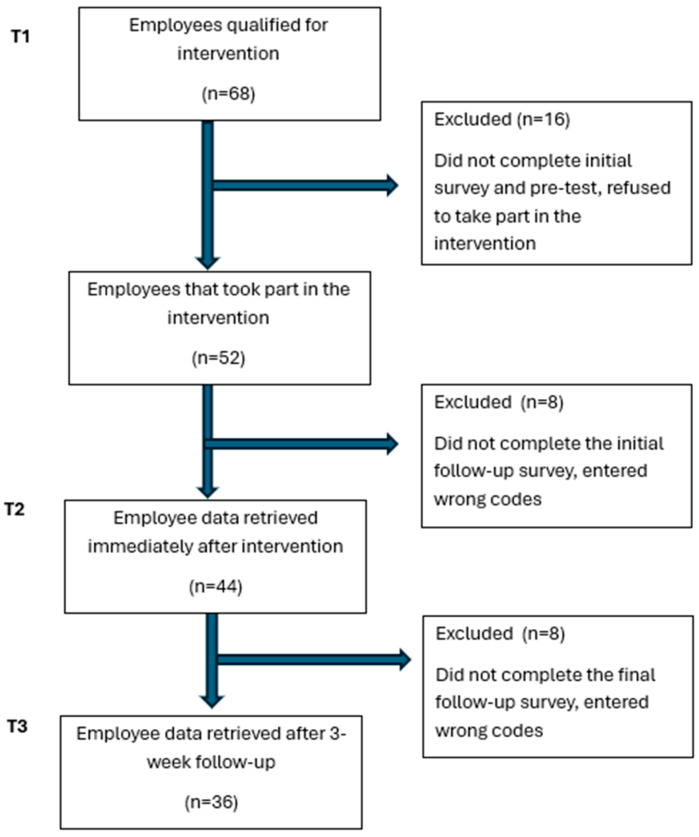
CONSORT flow adapted for a quasi-experimental study (*n* = 36). Abbreviations: T1—baseline; T2—post-intervention; T3—3-week follow-up; CONSORT—Consolidated Standards of Reporting Trials.

Figure 2 presents the participant flow using a CONSORT (Consolidated Standards of Reporting Trials) diagram adapted for quasi-experimental designs. Questionnaires were linked across timepoints (T1, T2, and T3) using individual identification codes generated by participants according to the researcher’s instructions and applied consistently at each measurement.

### 3.3. Intervention Description

The Brief Participatory Behavioral and Educational Workplace Intervention (BPBEWI) is an intervention designed to promote healthy eating in the workplace by reducing perceived dietary barriers and strengthening readiness to change dietary behaviors. The NLB approach (needs-based, learner-centered, behaviorally focused; [13]) was applied to identify employees’ dietary barriers prior to the intervention. The intervention incorporated behavioral economics strategies [4,11,12,26] aimed at facilitating healthier choices in the everyday work environment. Additionally, activities were designed to strengthen key psychological resources—self-efficacy, optimism, and resilience—that are known to positively influence the maintenance of health-promoting behaviors [15,17,19,20,27]. Table 1 presents the logic model outlining the relationship between inputs, mechanisms, and expected outcomes.

The intervention was conducted in the participants’ natural work environments during working hours, in rooms provided by the employer. The intervention was implemented in two public educational institutions located in the Warsaw metropolitan area, employing administrative and teaching staff. Both sites represented typical medium-sized schools, facilitating replication in similar occupational contexts. The employer provided organizational support (room, projector, access to water and healthy snacks). Participants were not required to travel to another facility, which minimized logistical barriers and enhanced the ecological validity of the intervention. Attendance did not interfere with routine professional responsibilities.

The intervention was implemented according to an identical protocol in both institutions, with a fixed sequence of components and consistent timing throughout. The facilitator used a checklist to ensure consistent delivery. During the session, participants worked with worksheets (see Supplementary S1 and S2). After the session (T2), they received a brochure (see Supplementary S3) containing simple recipes, strategies for preparing healthy meals, and examples of workplace lunch plans.

The 60 min intervention consisted of a single group session conducted in the participants’ workplace. It was an interactive, small-group workshop combining a brief lecture, guided discussion, and hands-on exercises. The intervention was divided into two parts: a 10 min theoretical component and a 50 min practical component. The theoretical part addressed the impact of workplace nutrition on cognitive functioning, mood, and productivity, as well as elements of behavioral economics in the context of dietary choices (default options, decision fatigue, heuristics). Examples of environmental nudges that support healthy choices were also presented. The practical part included three exercises. The first exercise, Lunchbox Building Blocks, involved assembling a healthy lunch from five categories of products (protein, complex carbohydrates, vegetables/fruits, healthy fats, and add-ons). This activity aimed to simplify the decision-making and establish default options for healthy workplace meals. The second exercise, Healthy Eating Plate, aimed to teach the intuitive evaluation of meal quality and the practical application of healthy eating principles. The task involved assessing and planning meals according to the Harvard Healthy Eating Plate model [28], which emphasizes visual proportions of food groups. Participants filled half of their plates with fruit and vegetables, one-quarter with whole grains, and one-quarter with healthy protein sources. They used healthy oils in moderation and chose water as the primary beverage. To enhance cultural relevance and understanding, the workshop incorporated the Polish adaptation of the Healthy Eating Plate, developed by the National Centre for Nutrition Education [29], which visually represents proportions of food groups adapted to national dietary guidelines and typical meal patterns.

The third exercise, Overcoming Barriers, centered on identifying barriers and developing strategies to overcome them. The session concluded with a Behavioral Call to Action, in which facilitators asked participants to write down at least one small change they could implement the next day (e.g., preparing a healthy lunch). This activity aimed to translate knowledge into immediate action and support habit formation.

The content and format of the practical exercises were designed to incorporate psychological mechanisms that draw on participants’ key resources, particularly self-efficacy (low-threshold tasks, opportunities for autonomous decision-making, incremental goals), resilience (collaborative problem-solving, sharing of experiences), and optimism (positive framing of messages, emphasizing the benefits of healthy choices).

Table 1 presents the structure, theoretical underpinnings, psychological targets, and verification methods of the intervention.

The intervention was conceptually grounded in the Behavior Change Technique (BCT) Taxonomy v1, as proposed by Michie et al. [22]. It integrated participatory, experiential, and self-regulatory strategies. Its behavioral logic followed an inputs–mechanisms–outcomes framework consistent with current implementation science and Self-Determination Theory (SDT) (see Table 2).

Inputs included a 60 min participatory workshop incorporating visual and practical tools (Healthy Eating Plate and Lunchbox Building Blocks exercises), as well as an educational brochure with simple workplace recipes.

The mechanisms and mediators aimed to enhance knowledge, self-efficacy, and autonomous motivation by employing several key BCTs: problem solving (BCT 1.2), action planning (BCT 1.4), instruction on how to perform the behavior (BCT 4.1), feedback on behavior (BCT 2.2), and verbal persuasion about capability (BCT 15.1). Group interaction provided additional social support (BCT 3.1), and the take-home brochure served as an environmental cue to sustain the behavior (adding objects to the environment, BCT 12.5) [22].

The expected outcomes were reduced perceived barriers, a strengthened sense of competence and control, and greater readiness to plan and prepare healthy meals for work. This logic model illustrates how participatory learning and self-regulatory techniques activate cognitive and motivational pathways that lead to behavioral intentions.

The content was tailored from the dietary barriers that employees self-reported in a pre-session needs assessment.

The intervention was delivered by an experienced dietetics and public health (A.H.) medical university graduate with a master’s degree and extensive experience in conducting health-related training and educational interventions. The facilitator had both subject-matter expertise and pedagogical competence to implement the intervention in line with behavioral intervention principles and the NLB approach. In addition, the content was reviewed by a psychologist (M.J.) and a public health specialist (I.C.).

Prior to implementation, the facilitator participated in a two-hour preparatory training session led by the coordinating psychologist (M.J.). The training included a review of the intervention protocol, a discussion of behavioral change techniques, and role-playing exercises to standardize the delivery style and ensure consistency across settings. A written manual and a facilitator checklist were provided to promote adherence to the protocol.

Throughout the implementation phase, the facilitator received ongoing supervision from the coordinating researcher (M.J.), who reviewed session documentation and provided feedback after the initial delivery. This ensured procedural consistency between institutions.

No major modifications were made during implementation, and both institutions followed the same protocol.

The implementation team monitored the intervention by documenting the sessions’ duration and progression. Attendance was recorded at each session, and all 36 participants completed the full 60 min workshop (*n* = 36). To ensure consistent implementation, the checklist included items related to timing, the sequence of exercises, facilitator prompts, and participant engagement. After each session, the coordinating psychologist (M.J.) reviewed the completed checklists and provided feedback to verify procedural fidelity. No major deviations from the standardized protocol were identified across sites. Upon completion, participants evaluated the intervention’s usefulness. Acceptability was assessed via a brief, anonymous, post-session questionnaire that evaluated perceived usefulness, relevance, satisfaction, and willingness to recommend the session to colleagues. Three weeks after the session (T3), the research teams administered an online follow-up to assess the maintenance of the short-term effects.

### 3.4. Primary and Secondary Outcomes

The primary outcome of this study was the change in the number of subjectively perceived dietary barriers between baseline (T1) and the three-week follow-up (T3).

Secondary outcomes included the following:

1. Maintenance of the declared intention to modify dietary behaviors three weeks after the intervention (T2 vs. T3).
2. Increase in subjectively perceived knowledge about healthy eating in the workplace (T2).
3. Subjective evaluation of the usefulness of the intervention on a 0–10 scale (T2).

### 3.5. Scientific Tools

This study employed a set of questionnaire tools covering sociodemographic, occupational, dietary, and psychological variables, as well as the perceive usefulness of the intervention. The instruments were administered in paper form (baseline—T1 and immediately post-intervention—T2) and electronically (three-week follow-up—T3).

At baseline (T1), data were collected on sociodemographic, occupational, dietary, and psychological variables, as along with perceived barriers to healthy eating at work. At T2, participants provided information regarding their intention to change and the perceived usefulness of the intervention, while at T3, data were collected on the maintenance of behavior changes adopted in the intervention.

#### 3.5.1. Sociodemographic, Occupational, and Dietary Data

We characterized participants in terms of basic sociodemographic variables (age, sex, educational level, and place of residence), occupational factors (type of employment, average weekly working hours, nature of work performed, and gross monthly income), and dietary habits. The dietary behavior questionnaire included questions regarding the number of meals consumed during working hours, the types of meals consumed (breakfast, lunch, snacks), preparation of meals for work (always, sometimes, never), and self-assessed nutrition knowledge (a 3-point descriptive scale).

#### 3.5.2. Workplace Healthy Eating Barriers Scale

The list of perceived barriers was developed through a structured process that combined research data and expert consensus. First, the research team identified potential items based on previous studies addressing barriers to meal preparation and dietary change in the workplace [4,5,6,7,8,9,10,11,12]. Then, a multidisciplinary research team (dietitian, psychologist, and public health specialist) reviewed them to ensure content validity and contextual relevance for the school workplace setting. The final list included six categories of barriers: lack of time, lack of knowledge, lack of motivation, lack of appropriate ingredients, unwillingness to cook, and other (open-ended).

Participants could select more than one barrier. Additionally, the participants were asked whether they perceived preparing healthy meals as time-consuming, rated on a 4-point scale ranging from “definitely yes” to “definitely no”.

#### 3.5.3. Psychological Resources

Standardized instruments in their Polish adaptations were used to measure psychological variables.

The Generalized Self-Efficacy Scale (GSES) developed by Schwarzer and Jerusalem and adapted by Juczyński consists of 10 items rated on a 4-point scale (1–4). It measures an individual’s belief in their ability to cope with difficult situations and achieve intended goals. The total score ranges from 10 to 40 points, with higher scores indicating greater self-efficacy. In the present study, the internal consistency of the Polish version was satisfactory (Cronbach’s α = 0.85), consistent with previous validation research. The GSES has demonstrated good reliability and construct validity, showing positive associations with adaptive coping, self-control, and proactive health behaviors [32].

The Life Orientation Test-Revised (LOT-R) developed by Scheier, Carver, and Bridges, adapted by Poprawa, contains 10 items (6 diagnostic, 4 fillers) that are rated on a 5-point scale (0–4). The test measures dispositional optimism, which is defined as the tendency to expect positive events in the future. The overall score is the sum of the diagnostic items, and researchers code the responses to the negatively worded items in reverse. In this study, the Polish version demonstrated acceptable internal consistency (Cronbach’s α = 0.76). The LOT-R has demonstrated adequate reliability, factorial validity, and strong criterion validity, correlating with lower stress and higher subjective well-being [32].

The Brief Resilient Coping Scale (BRCS), developed by Sinclair and Wallston and adapted by Piórowska et al., is a 4-item scale rated on a 5-point Likert scale. It measures an individual’s capacity for resilient coping in challenging situations. Total scores range from 4 to 20, with higher scores reflecting greater resilience. In the present sample, the internal consistency was modest (Cronbach’s α = 0.63), which is typical of very short scales. The BRCS demonstrates satisfactory convergent validity [33].

#### 3.5.4. Assessment of Intent to Change and Intervention Usefulness

Immediately after the intervention (T2), participants responded to “yes/no” questions regarding their intention to prepare healthy meals for work, their intention to change their eating habits, and their perception of increased nutrition knowledge. Additionally, participants rated the usefulness of the session on a 0–10 scale (0 = “not useful at all”, 10 = “very useful”).

#### 3.5.5. Assessment of Effect Maintenance at Three-Week Follow-Up

At three-week follow-up (T3), participants completed an online questionnaire that included the following items: barriers they had overcome, perceived ease of preparing healthy meals for work, motivation stability to prepare healthy meals for work, meal planning regularity for work, culinary skills development for preparing healthy meals for work, and change durability in dietary behaviors. The questions were categorical or ordinal, and researchers tailored them to the content of each topic.

### 3.6. Statistical Analysis

All statistical analyses were conducted using IBM SPSS Statistics (version 14). The significance level was set at *p* < 0.05 for all tests. Descriptive analyses were first performed to obtain statistics on demographic variables, dietary behaviors, and psychological characteristics. We presented categorical variables as frequencies and percentages, and continuous variables using means, standard deviations, and ranges.

Analyses were performed using a complete-case approach, including only participants who provided data at all three measurement points (T1–T3). Missing data were minimal and primarily occurred due to incomplete responses in the online three-week follow-up questionnaire (T3). Since the missing data were related to practical or organizational factors (e.g., workload, limited access to a computer during the follow-up week) rather than participant characteristics or study variables, we assumed the data to be missing at random.

Due to the exploratory and pilot nature of this study, the research team did not conduct a sample size calculation a priori. The total number of eligible employees available at the time of the intervention in both participating schools served as the sample size (*n* = 36). This approach aligns with recommendations for feasibility and pilot studies that focus on testing procedures, acceptability, and short-term effects rather than formal hypothesis testing.

To provide additional transparency, the researchers performed a post hoc power analysis for the primary outcome (reduction in perceived dietary barriers, paired Wilcoxon signed-rank test). For the observed large effect size (r = 0.78; α = 0.05), the achieved statistical power was 1 − β = 0.94. While this indicates adequate sensitivity to detect large effects, the small sample size limits the external validity and generalizability of the findings. Therefore, the results should be interpreted as preliminary evidence supporting the feasibility and short-term potential of the intervention.

To evaluate the effectiveness of the intervention in reducing dietary barriers, we compared the number of barriers reported by participants at baseline (T1) and at the three-week follow-up (T3). Since the data distribution deviated from normality, the team performed a paired Wilcoxon signed-rank test, and they reported effect sizes as the coefficient *r* (Z/√N) and the rank–biserial correlation (*rb*). For paired binary responses (Yes/No), changes between the baseline and post-intervention were analyzed using McNemar’s test, and effect sizes were expressed as the risk difference (RD) and odds ratio (OR). For continuous outcomes, such as satisfaction with the workshop (0–10 scale), the effect size was calculated using Cohen’s *d*.

Due to logistical constraints and the exploratory nature of this study, the researchers did not conduct a priori power analysis. The interpretation of results should therefore be cautious and considered as a preliminary validation of the intervention model.

### 3.7. Ethics and Informed Consent

The study team informed all participants about the purpose of this study and that their participation was voluntary. Each participant signed a written informed consent form that included permission for the processing of anonymized data for scientific purposes. The Ethics Committee of the Medical University of Warsaw approved this study (approval no. AKBE/291/2023, 9 October 2023), confirming its compliance with the current ethical standards for research involving human participants. We collected and analyzed data in a manner that ensured anonymity and confidentiality.

## 4. Results

### 4.1. Participants

All participants had completed higher education, and 100% were teachers or administrative staff. The mean age was 46.81 years (SD = 11.95); the youngest participant was 24 years old, and the oldest was 64 years old. The group included 30 women (83.3%) and 6 men (16.7%). All participants were employed under a work contract, and most worked on-site five days a week (91.70%; *n* = 33). One participant reported working four days per week (2.8%), while two reported two on-site days per week (5.6%). Participants reported an average of 35.39 h worked per week (SD = 13.92), ranging from 6 to 70 h (no shift work).

The survey asked participants to indicate their gross monthly income bracket. The most frequently selected range was PLN 6000 to 7999 (*n* = 22; 61.1%). Other responses were distributed as follows: PLN 4242 to 5999 (*n* = 7; 19.4%), PLN 8000 to 9999 (*n* = 5; 13.9%), below PLN 4242 (*n* = 1; 2.8%), and above PLN 10,000 (*n* = 1; 2.8%).

The mean self-efficacy level was 29.06, with moderate variability (SD = 4.51). Participants demonstrated relatively high levels of optimism (M = 15.06; SD = 4.08) and psychological resilience (M = 14.39; SD = 3.06) (see Table 3).

Most participants had one or two meals at work, primarily snacks and breakfasts. Lunches were reported much less frequently. Some participants prepared their own meals, though few did so regularly. In contrast, the vast majority reported eating breakfast before the start of the workday (Table 4). All employees had equal access to the same on-site cafeteria, which offered the same meal options to school staff and students.

### 4.2. Baseline Barriers and Perceptions

Only three participants reported experiencing none of the barriers listed for preparing healthy meals for work. The remaining participants identified one (*n* = 17; 47.2%), two (*n* = 14; 38.9%), or three (*n* = 2; 5.6%) barriers. The results (see Table 5) indicate that lack of time was the most frequently reported barrier.

Of the 36 study participants, 5 individuals (13.9%) indicated that preparing healthy meals for work is “definitely” time-consuming, and 19 individuals (52.8%) stated that it is “rather” time-consuming. In contrast, 12 participants (33.3%) expressed the opposite view, selecting “not rather”. No participants chose “definitely not” or “hard to say”.

### 4.3. Primary Outcome: Changes in Perceived Barriers Following the Intervention

The paired Wilcoxon signed-rank test (n = 36) revealed a significant reduction (T1 vs. T3) in the number of dietary barriers following the intervention (Δ = −1 [IQR: −2 to 0]; Z = −4.69, *p* < 0.001, r = −0.78). These findings imply that the intervention substantial decreased the number of perceived obstacles to health-promoting behaviors in the workplace (see Table 6).

### 4.4. Immediate Post-Intervention Outcomes (T2)

At baseline (T1), 25.00% of participants (*n* = 9) reported consistently adhering to healthy eating principles. Meanwhile, 63.90% (*n* = 23) were aware of the recommendations, yet did not always implement them, and 11.10% (*n* = 4) indicated gaps in their knowledge. Immediately post-intervention (T2), the majority (86.1%, *n* = 31) reported an increase in knowledge about healthy eating in the workplace, while 13.9% (*n* = 5) reported no change. McNemar’s test revealed a statistically significant increase in the proportion of participants who perceived an improvement in their nutrition-related knowledge following the workshop (*p* < 0.001; OR = 23.00, 95% CI: 3.10–170.10; risk difference = +61.1 percentage points).

To assess the immediate post-intervention behavioral intentions (T2), participants were asked if they planned to alter their eating habits or prepare healthy meals for work. The proportion of participants intending to change their eating habits increased significantly after the intervention, rising from 36.1% (*n* = 13) at baseline (T1) to 86.1% (*n* = 31 at the three-week follow-up (T3). McNemar’s test confirmed a significant improvement in readiness to change (*p* < 0.001; OR = 10.00, 95% CI: 1.80–54.50; risk difference = +50.0 percentage points). Notably, a high proportion of participants (91.7%; *n* = 33) declared an intention to prepare healthy meals for work. McNemar’s test confirmed a statistically significant increase in the behavioral intention to prepare healthy meals for work (*p* < 0.001; OR = 22.00, 95% CI: 2.90–165.50; risk difference = +58.3 percentage points), indicating a very large effect size.

The subjective evaluation of the intervention used a 1 to 10 scale (1 = “not useful at all”, 10 = “very useful”). The mean rating was 8.28 (SD = 1.77), with responses ranging from 3 to 10 (*n* = 36). These results indicate a high level of acceptance of and satisfaction with the intervention. Only two participants gave low ratings (3 and 4) but did not provide comments justifying their evaluations. Additional analyses showed a very large effect size (Cohen’s *d* = 1.85), confirming that participants perceived the intervention as highly valuable.

### 4.5. Sustained Effects at Three-Week Follow-Up (T3)

At three-week follow-up (T3), the majority of the participants reported continued positive effects (see Table 7). A large proportion (86.1%) indicated that preparing healthy meals for work was easy and they were highly or moderately motivated to do so. Over 80% of the participants said that they always or sometimes planned healthy meals for work following the intervention. However, the intervention did not clearly improve culinary skills. Some participants stated that their abilities had not changed and still required practice, though they reported increased motivation to prepare healthy lunches. Over 90% of the participants reported maintaining at least one dietary change during the three weeks after the intervention.

No adverse events or unintended effects were reported during the intervention.

### 4.6. Summary of Primary and Secondary Outcomes

The overall effects of the intervention across all primary and secondary outcomes are summarized in Table 8, which presents pre- and post-intervention values, changes (Δ), statistical tests, and effect sizes with 95% confidence intervals.

## 5. Discussion

### 5.1. Summary of Main Findings

This study primarily aimed to evaluate the short-term impact of a BPBEWI conducted in a school setting among white-collar employees (teachers and administrative staff) on perceived dietary barriers and participants’ readiness to change dietary behaviors. The intervention was associated with a reduction in reported barriers, which provides preliminary support for Hypothesis H1, suggesting that targeted educational and behavioral support could be useful for this occupational group.

Regarding the secondary outcomes, the participants reported an increase in their perceived nutritional knowledge, and a strong intention to improve their dietary habits. They also reported a very high intention to prepare healthy meals for work. The participants also rated the intervention as highly useful, reflecting their high satisfaction with the content and format of the sessions (H2).

At three-week follow-up (T3), most participants reported that they had maintained positive changes. These changes included reduced perceived difficulty in meal preparation, sustained motivation, and consistent meal planning for work. A large proportion of participants reported having made at least one specific change to their eating habits. These results provide preliminarily support for Hypothesis H3, which suggests the short-term maintenance of reported behavioral intentions following the intervention. However, the lack of long-term follow-up limits our ability to draw conclusions about the durability of the changes.

### 5.2. Interpretation in Relation to Prior Research

The findings align with the growing interest in the feasibility and potential benefits of short-term environmental and behavioral interventions in the workplace [5,34]. The reduction in perceived dietary barriers after participating in a BPBEWI is consistent with previous reports indicating that even single-session, well-designed educational and motivational initiatives can reshape the perception of difficulties associated with healthy eating [5,35]. This is particularly true for behavioral barriers, such as a lack of a meal preparation habits, difficulty with planning, and an absence of practical solutions.

Demonstrating simple strategies, modeling behaviors, and providing opportunities to practice specific solutions (e.g., lunch planning) may foster behavioral changes more readily than cognitive education alone. Unlike deeply held beliefs or values, behavioral barriers often stem from a lack of experience or routine-based actions and may be more susceptible to change through brief, well-structured interventions [35].

The intervention was designed to influence the automatic decision-making processes related to food choices. It employed simplified planning tools and default action schemes, such as the Lunchbox Building Blocks exercise. This exercise drew on behavioral economics mechanisms, including decision fatigue, limited cognitive capacity, and the tendency to maintain the status quo [6,11]. By reducing the cognitive load associated with meal planning, the researchers encouraged participants to make healthier choices independently.

At the same time, the practical exercises, such as Lunchbox Building Blocks and Healthy Eating Plate, allowed participants to immediately apply their knowledge, develop meal-planning skills, and visualize nutrient proportions. Thus, the intervention integrated a practical and an educational component, activating self-regulation processes (action planning, anticipating difficulties) and strengthening the sense of competence and autonomy [21,22,36]. According to Self-Determination Theory [21,37], these are key components of intrinsic motivation.

From a psychological perspective, implementing a simple and feasible strategy seemed to minimize the perceived barriers, which often play a greater role in readiness to act than actual resource constraints. When barriers are perceived as overwhelming, individuals may abandon attempts at change, even if they have the means to do so objectively. Reducing the subjective burden of these barriers may help restore a sense of agency and initiate action, which helps explain the study findings [38,39,40].

The observed outcomes may have been influenced three key psychological resources of the participants: self-efficacy, optimism, and resilient coping [41,42]. The designed practical activities allowed participants to experience agency in meal planning, which strengthened self-efficacy. These activities introduced simple, attainable strategies that fostered positive expectations about the success of change, promoting optimism. The activities also encouraged the search for feasible solutions within a demanding work environment, developing resilient coping. Consequently, the intervention appears to have addressed cognitive, practical, and psychological barriers to change readiness.

The intervention included a brochure with quick, simple, and healthy recipes for workplace meals, designed for everyday professional settings. Including the brochure may have positively influenced participant engagement and strengthened their readiness to implement change. As demonstrated in the study by Moreau et al., providing participants with simple, practical materials (e.g., recipes, dietary strategies) can enhance competence and facilitate the adoption of healthy habits, especially in daily work life [43].

From a psychological perspective, the brochure served as a tool that supported autonomy and competence, which are two key components of intrinsic motivation according to Self-Determination Theory [21,37]. Additionally, it may have reduced anxiety related to failure or a lack of ideas (“What can I actually eat?”), which often poses a barrier to adopting healthy habits. In the context of a one-time intervention of this type, well-designed, practical materials may be an important element in supporting the transfer of knowledge from the workshop setting to participants’ everyday professional lives [42].

The three-week follow-up results (T3) are consistent with these observations. Most participants reported maintaining changes, increased motivation, and greater ease in meal preparation. The literature emphasizes that brief interventions can initiate change when they address participants’ specific needs and are embedded in their daily lives [5]. Participants’ ability to maintain changes independently, without further structural support, suggests the successful activation of internal motivational and cognitive mechanisms.

Beyond these individual-level mechanisms, contextual and environmental factors may have also played a role in shaping the outcomes. The intervention was implemented in schools, where employees often have limited time for meals, short or fragmented breaks, and limited access to healthy food options in cafeterias [7,8,9]. These workplace constraints may have influenced perceived barriers and participants’ ability to apply the strategies introduced during the workshop. Additionally, external factors such as household responsibilities, caregiving duties, and financial constraints may have affected participants’ ability to plan and prepare healthy meals for work [44]. Furthermore, workplace conditions, such as the fixed duration of lunch breaks and the limited cafeteria meal options or pricing, may have further constrained behavioral change. Although these contextual influences were not formally assessed, they may have moderated the observed outcomes and should be systematically examined in future controlled studies.

### 5.3. Implications for Practice

The study findings offer valuable insights for practitioners of workplace health promotion. They demonstrate that a brief, one-session intervention can effectively reduce perceived barriers and enhance readiness to make healthy dietary choices. This suggests that health promotion initiatives do not always need to be long-term or intensive; rather, they should focus on real challenges and adapt to everyday working conditions.

From a practical perspective, it is crucial to focus on real barriers rather than normative messaging alone. An approach consistent with motivational interviewing is to work with barriers through identification, reframing, and collaborative problem-solving [45]. This approach posits that lasting behavior change is more likely to stem from personal reflection and intrinsic motivation than from external directives. Similarly, the Solution-Focused Approach (SFA) [46] does not emphasize analyzing the causes of unhealthy behaviors. Instead, it focuses on identifying resources, finding feasible strategies, and strengthening the belief in the possibility of change. This approach fostered an increased sense of agency and motivation, and it allowed for the individualization of the workshop experience despite its group format.

Including short nutrition modules within existing structures, such as occupational health and safety (OHS) or corporate social responsibility (CSR) initiatives, is particularly valuable. This approach increases the appeal and ease of implementing interventions within organizations, because they become part of mandatory or strategic employer activities.

The intervention model used in this study can be adapted to various sectors, including education, public administration, services, and small businesses. Minimal organizational requirements include a 60 min workshop, supporting materials (e.g., brochures, simple planning tools), and a professional trained in dietetics or health promotion to deliver it. Due to its low cost and flexibility, this model is easily scalable and adaptable to the specific needs of different occupational groups.

### 5.4. Strengths and Limitations

This study has several strengths that enhance its practical value and potential applications. First, the research team designed and implemented the intervention in a natural work environment, where it accounted for the actual needs and circumstances of the participants. Importantly, this study is among the few that focus on white-collar school employees, a professional group rarely included in nutritional interventions despite their high cognitive demands and limited opportunities for meal breaks. The applied model, a BPBEWI, proved feasible with minimal organizational resources, and participants evaluated it positively. Its participatory and problem-oriented structure deserves particular attention. It enabled participants to actively engage in identifying and analyzing dietary barriers, rather than limiting the process to passive education. Another strength of this study was the inclusion of a three-week follow-up (T3), which allowed for a preliminary assessment of effect sustainability. Finally, the integration of educational and behavioral elements with low-threshold psychological components makes this intervention easy to replicate and adapt in other occupational settings.

However, despite these advantages, this study has important limitations. First, it used a pre–post design without a control group, which substantially limits internal validity and prevents causal inferences. The observed changes may have resulted from external factors (e.g., workplace circumstances) or participant expectations. Additionally, the possibility of a Hawthorne effect must be considered, whereby improvements arise solely from participation in this study and increased attention rather than from the actual impact of the intervention. Second, the sample size was small (n = 36) and limited to individuals with higher education who worked in a large city. This limitation hinders the generalizability of the findings. Furthermore, this study did not address attrition, i.e., potential differences between participants who remained in this study and those who withdrew or were lost to follow-up. The absence of such analysis may limit the ability to fully assess the reliability of the results.

Another limitation is that the main variables were operationalized using self-report measures such as perceived knowledge, motivation, and declared behavioral changes. These variables are susceptible to bias (e.g., expectancy effects, social desirability), particularly in the absence of objective indicators (e.g., actual food intake, meal diaries, or biochemical markers).

It should be noted that the intervention was evaluated only twice: immediately after completion and at the three-week follow-up. Thus, long-term tracking, which would have allowed verification of the durability of the reported changes, was lacking. Moreover, the follow-up assessment relied solely on subjective self-reports rather than actual behavioral data. To minimize participant burden during a short workplace session, the researchers did not include a formal knowledge test; however, this limits the ability to quantify learning outcomes.

Another limitation is the issue of multiple comparisons. Since several related outcomes were analyzed, the probability of a Type I error cannot be excluded. No formal correction for multiple testing (e.g., Bonferroni or false discovery rate adjustment) was applied, because this study was designed as a pilot feasibility assessment. Therefore, secondary findings should be interpreted as exploratory, serving to identify potential directions and mechanisms to be verified in larger, controlled studies.

Given the small pilot sample and single baseline measurement of psychological resources, no exploratory analyses of their associations with changes in outcomes were conducted; this remains an important direction for future controlled studies with adequate statistical power.

### 5.5. Future Directions

The findings of this study suggest that brief, targeted workplace interventions can effectively reduce perceived dietary barriers and strengthen health-promoting intentions. However, future research should expand the scope of the analysis and use more advanced study designs to better understand the mechanisms of action and sustainability of such interventions.

Future studies should incorporate an objective nutrition knowledge test (8–12 multiple-choice or true/false items) assessing topics such as balanced meal composition, food labeling, and portion size estimation. Moreover, researchers plan to implement extended follow-up periods (8–12 weeks) to capture observable behavioral outcomes, such as the frequency of bringing lunch from home, cafeteria choices, and proxies for overall diet quality. This approach will allow for a more comprehensive assessment of the sustainability of behavioral changes.

First, researchers should use controlled designs to allow for causal inferences and assessment of intervention effectiveness compared to other forms of support (e.g., traditional health education or digital interventions). Including long-term measurements (e.g., at 3 and 6 months) would also be valuable for evaluating the durability of the reported changes and their translation into actual behaviors.

Second, an important area for future development is conducting an in-depth analysis of participants’ psychological profiles and their potential moderating role. Including variables such as the locus of control, coping style, and level of self-regulation may help identify groups that respond particularly well (or poorly) to a given type of intervention.

Another worthwhile area of exploration is testing different variants of the same intervention. For example, one could test individual formats, digital (online) versions, versions extended with coaching elements, and versions implemented in various occupational sectors (e.g., services, education, and public administration). Comparative studies could reveal which components (e.g., practical exercises, supporting materials, workshop format) are critical to effectiveness.

Finally, future projects should incorporate a more diverse set of effectiveness indicators. This set should encompass not only self-assessed knowledge or intentions but also objective data on dietary behaviors (e.g., food diaries, purchasing data, anthropometric measurements). This data could validate reported effects and provide robust evidence of the effectiveness of environmental nutrition interventions.

## 6. Conclusions

This pilot study provides preliminary evidence that a BPBEWI is feasible and acceptable, and potentially an effective approach to support employees’ dietary decision-making in the workplace. The intervention was associated with reduced perceived barriers, strengthened intentions toward healthier eating, and positive short-term feedback from participants.

However, due to the single-group pre–post design, reliance on self-reported measures, and the brief follow-up period after the immediate post-test, the findings should be interpreted as exploratory indicators of feasibility and potential usefulness rather than as proof of effectiveness.

From a practical perspective, these brief, participatory sessions may be a time-efficient and low-cost way for workplaces to promote healthy eating habits without disrupting routines. Integrating brief behavioral sessions into employee wellness or health promotion programs could increase awareness of, motivation for, and confidence in meal preparation and healthy food choices.

Further research employing controlled designs, objective outcome measures, and longer follow-up periods is necessary to confirm these preliminary findings and determine the intervention’s true impact on dietary behaviors and related health outcomes.

## Figures and Tables

**Table 1:** Structure, theoretical basis, and verification of the behavioral nutrition intervention based on the NLB approach.

Segment of the Intervention	Intervention Goals and Theoretical Basis	Model	Actions Supporting Psychological Resources (Self-Efficacy, Optimism, Resilience)	Methods of Verifying Goal Attainment	Duration
Introduction	Establish relevance for behavioral change; present evidence on the impact of workplace nutrition on cognitive function, mood, and productivity; introduce key behavioral economics principles (default options, decision fatigue, heuristics) and examples of workplace “nudges”.	Behavioral Economics [26], Situational Optimism [16]	Positive message framing; minimizing fear of failure; use of engaging examples; emphasis on benefits of small environmental changes	Active engagement, verbal reflection	10 min
Practical Exercise 1: “Lunchbox Building Blocks”	Simplify decision-making and promote creation of healthy default lunch options; increase planning skills and nutrition knowledge.	NLB approach [13], Choice Architecture [26], Bandura’s Self-Efficacy Theory [14]	Low-barrier task with immediate mastery experience; autonomy in food selection; visual guidance through five product categories (protein, complex carbohydrates, fruit/vegetables, healthy fats, additions)	Post-intervention readiness-to-change questionnaire; pre–post comparison of perceived barriers; 3-week follow-up behavioral analysis	20 min
Practical Exercise 2: “Healthy Eating Plate”	Develop intuitive skills for assessing and planning healthy meals using the Harvard Healthy Eating Plate model, emphasizing balanced proportions of food groups (½ fruit and vegetables, ¼ whole grains, ¼ healthy protein)	NLB approach [13], Healthy Eating Plate Guidelines [28,29]	Peer discussion; shared evaluation of meal examples; autonomy in creating balanced meals	Pre–post evaluation of nutrition knowledge; facilitator qualitative notes	15 min
Practical Exercise 3: Overcoming Barriers	Identify and reframe personal barriers to healthy eating at work; generate coping strategies and foster agency in addressing them.	NLB approach [13], Cognitive Load Theory [30]	Normalization of challenges; collective problem solving; peer-generated strategies	Pre–post comparison of barrier checklists; qualitative discussion notes	10 min
Behavioral Call to Action	Translate session content into immediate action; encourage commitment to at least one small change starting the next day (e.g., preparing a healthy lunch).	NLB approach [13], Small Wins Theory [31], Bandura’s Self-Efficacy Theory [14]	Verbal and written goal commitment; public sharing to reinforce accountability	Participant intention statements; readiness-to-change indicators	5 min

Note: Description of the BPBEWI implemented in two schools (*n* = 36). Each segment integrates theoretical foundations (needs-based, learner-centered, and behaviorally focused [NLB] approach; Behavioral Economics; Self-Efficacy Theory; Small Wins Theory), psychological resource targets (self-efficacy, optimism, resilience), and verification methods. Session duration: 60 min; one group session conducted at the workplace. Abbreviations: NLB—needs-based, learner-centered, and behaviorally focused approach; min—minutes.

**Table 2:** Mapping of intervention components to Behavior Change Techniques (BCTs) and corresponding psychological mechanisms.

Intervention Component	Mapped BCT (v1)	Psychological Mechanism/Mediator	Expected Outcome
Identification of personal dietary barriers	Problem solving (BCT 1.2)	Awareness of obstacles; cognitive reframing	Reduced perceived barriers
“Lunchbox Building Blocks” exercise (planning balanced meals)	Action planning (BCT 1.4)	Concrete goal formation; increased perceived control	Improved meal-preparation intentions
“Healthy Eating Plate” visual task (portion composition)	Instruction on how to perform the behavior (BCT 4.1)	Procedural knowledge; visualization of healthy proportions	Enhanced nutrition-related knowledge
Group discussion and facilitator feedback	Feedback on behavior (BCT 2.2); social support (unspecified) (BCT 3.1)	Social modeling; self-evaluation; relatedness	Increased motivation and engagement
Encouragement and reinforcement from facilitator	Verbal persuasion about capability (BCT 15.1)	Strengthened self-efficacy and optimism	Higher readiness for change
Educational brochure with recipes and strategies	Adding objects to the environment (BCT 12.5)	Environmental cue for autonomous continuation	Maintenance of self-directed behavior

**Table 3:** Descriptive statistics for psychological variables: self-efficacy, resilience, and optimism.

Psychological Variable	Min.	Max.	M	SD	Variable Intensity Level (*)		
					Low *n* (%)	Medium *n* (%)	High *n* (%)
Self-efficacy	20	40	29.06	4.510	5 (13.90)	14 (38.90)	17 (47.20)
Resilience	6	20	14.39	3.064	10 (27.80)	15 (41.70)	11 (30.60)
Optimism	6	22	15.06	4.084	10 (27.80)	14 (38.90)	12 (33.30)

Data represent baseline (T1) results of participants who completed all measurement points (*n* = 36). Levels categorized according to Polish population norms (*) [32,33]. Note: *n*—number of participants; M—mean; SD—standard deviation; Min.—minimum; Max.—maximum; %—percentage of participants. Measurement tools: GSES—Generalized Self-Efficacy Scale; BRCS—Brief Resilient Coping Scale; LOT-R—Life Orientation Test-Revised.

**Table 4:** Dietary behaviors during work hours, baseline (T1).

Variable		*n*	%
Number of meals consumed at work	0	1	2.80
	1	11	30.60
	2	19	52.80
	3	5	13.90
Breakfast consumption at work	yes	22	61.10
	no	14	38.90
Lunch consumption at work	yes	14	38.90
	no	22	61.10
Snack consumption at work	yes	28	77.80
	no	8	22.20
Self-preparation of meals for work	no	11	30.60
	yes, sometimes	12	33.30
	yes, always	13	36.10

Note: Frequencies and percentages of meal-related behaviors during work hours at baseline (T1). Variables include number and type of meals consumed at work and frequency of meal preparation. Abbreviations: *n*—number of participants; %—percentage.

**Table 5:** Self-reported barriers to preparing healthy meals for work (*n* = 36).

Barriers to Preparing Healthy Meals for Work	Number of Responses (*n*)	Percentage (%)
Lack of time at work to eat a healthy meal	29	80.60
Insufficient knowledge	0	0
Unavailability of appropriate ingredients	8	22.20
Lack of motivation	6	16.70
Reluctance to cook	7	19.40
Other—excessive workload	1	2.80

Note: Participants could indicate more than one barrier. Results expressed as number and percentage of responses. Abbreviations: *n*—number of responses; %—percentage. Data collected at baseline (T1).

**Table 6:** Change in the number of reported barriers at baseline (T1) and at three-week follow-up (T3)—the paired Wilcoxon test (*n* = 36).

Type of Change	Number of Cases (*n*)	Mean Rank	Sum of Ranks
Reduction in the number of barriers (negative ranks)	26	13.50	351.00
Increase in the number of barriers (positive ranks)	0	0	0
No change (ties)	10	–	–

Detailed results for individual perceived barriers. Summary statistics are presented in Table 7. Note: The analysis compares the number of perceived dietary barriers at baseline (T1) and at three-week follow-up (T3). Test: Paired Wilcoxon signed-rank test (Z = −4.689; *p* < 0.001; r = 0.78). Abbreviations: *n*—number of participants; M—mean; SD—standard deviation; Z—test statistic; *p*—significance level; r—effect size calculated as Z/√N.

**Table 7:** Self-reported changes at three-week follow-up (T3) (*n* = 36).

Variable		*n*	%
Perceived ease of preparing healthy meals for work	Definitely no	1	2.80
	Rather no	4	11.10
	Rather yes	27	75.00
	Definitely yes	4	11.10
Stability of motivation to prepare healthy meals for work	Low	8	13.90
	Moderate	18	50.00
	High	13	36.10
Regularity of planning meals for work	Never	2	5.60
	Sometimes	23	63.90
	Rarely	4	11.10
	Always	7	19.40
Development of culinary skills in preparing healthy meals for work	No, my skills have not changed	5	13.90
	Not really, but I feel more motivated	14	38.90
	Yes, but I still need more practice	15	41.70
	Yes, definitely	2	5.60
Durability of introduced dietary changes	Yes	33	91.70
	No	3	8.30

Note: Percentages of participants reporting maintenance of positive dietary behaviors at three-week follow-up (T3). Variables: Perceived ease of preparing healthy meals, motivation stability, meal planning regularity, culinary skill development, and durability of dietary changes. Abbreviations: *n*—number of participants; %—percentage.

**Table 8:** Primary and secondary outcomes.

Outcome	T1	T2	Δ (Change)	Test	*p*	Effect Size [95% CI]
Primary outcome						
Perceived dietary barriers (total score)	Median = 1.0 [IQR: 1–2]	Median = 0 [IQR: 0–1]	−1 [IQR: −2 to 0]	Paired Wilcoxon Z = −4.69	<0.001	r = −0.78
Secondary outcomes						
Intention to change eating habits (Yes, %)	36.10%	86.10%	+50.0 pp	McNemar χ^2^(1) = 13.14	<0.001	OR = 10.00 [1.80–54.50]
Intention to prepare healthy meals for work (Yes, %)	33.30%	91.70%	+58.3 pp	McNemar χ^2^(1) = 17.39	<0.001	OR = 22.00 [2.90–165.50]
Nutrition-related knowledge (Yes, %)	25.00%	86.10%	+61.6 pp	McNemar χ^2^(1) = 21.7	<0.001	OR = 23.00 [3.10–170.10]
Workshop satisfaction (0–10 scale)	-	Mean = 8.28 (SD = 1.15)	-	-	-	Cohen’s *d* = 1.98

Note: Effect sizes and confidence intervals are reported for each test. McNemar’s test was applied to paired binary outcomes, and the paired Wilcoxon signed-rank test to paired ordinal data. Significance threshold was set at *p* < 0.05 (two-tailed). Abbreviations: T1—baseline (pre-intervention); T2—post-intervention; Δ (Change)—difference between T2 and T1; IQR—interquartile range; pp—percentage points; Z—the paired Wilcoxon signed-rank test statistic; χ^2^(1)—McNemar’s chi-square test with 1 degree of freedom; OR—odds ratio; CI—confidence interval; r—effect size for paired Wilcoxon test (rank–biserial correlation); Cohen’s *d*—standardized mean difference (effect size).

## Data Availability

The original data presented in this study are available in the Supplementary Materials attached to this article. The anonymized datasets used in this study are publicly available in the Open Science Framework (OSF) repository at https://osf.io/mrh2n/ (assessed on 20 October 2025).

## Supplementary Materials

The following supporting information can be downloaded at https://www.mdpi.com/article/10.3390/nu17213371/s1, Supplementary Materials included a handout that the participants worked on during the intervention (Supplementary S1 and Supplementary S2), an information brochure distributed to workshop participants containing simple recipes (Supplementary S3), TREND checklist final and BPBEWI data.

## Abbreviations

The following abbreviations are used in this manuscript:

WHO	World Health Organization
OECD	Organization for Economic Co-operation and Development
GDP	Gross Domestic Product
NLB approach	Needs-based, learner-centered, and behaviorally focused approach
TREND	Transparent Reporting of Evaluations with Nonrandomized Designs
T1	Baseline
T2	Immediately post-intervention
T3	Three-week follow-up
GSES	Generalized Self-Efficacy Scale
LOT-R	Life Orientation Test-Revised
BRCS	Brief Resilient Coping Scale
N	Number of responses
SD	Standard deviation
M	Mean
Min.	Minimum
Max.	Maximum
Z	Wilcoxon signed-rank test
*p*	Significance level
RD	Risk difference
CI	Confidence interval
OR	Odds ratio
h	Cohen’s h
pp	Percentage points
SFA	Solution-Focused Approach
OHS	Occupational health and safety initiatives
CSR	Corporate social responsibility initiatives
SDT	Self-Determination Theory
CBTs	Cognitive–Behavioral Techniques
TIDieR	Template for Intervention Description and Replication
COM-B	Capability, Opportunity, Motivation → Behavior
CONSORT	Consolidated Standards of Reporting Trials
BCT	Behavior Change Technique
BPBEWI	Brief Participatory Behavioral and Educational Workplace Intervention
