# Supplementary material for: A Brief Participatory Workplace Intervention on Dietary Barriers and Healthy Eating Intentions Among Employees: A Pilot Study
Source: Nutrients. 2025 Oct 27;17(21):3371. doi: 10.3390/nu17213371 (PMC12610673; doi:10.3390/nu17213371)
Supplement: Supplementary file 1 [file nutrients-17-03371-s001.zip › Suplementary S3.pdf]

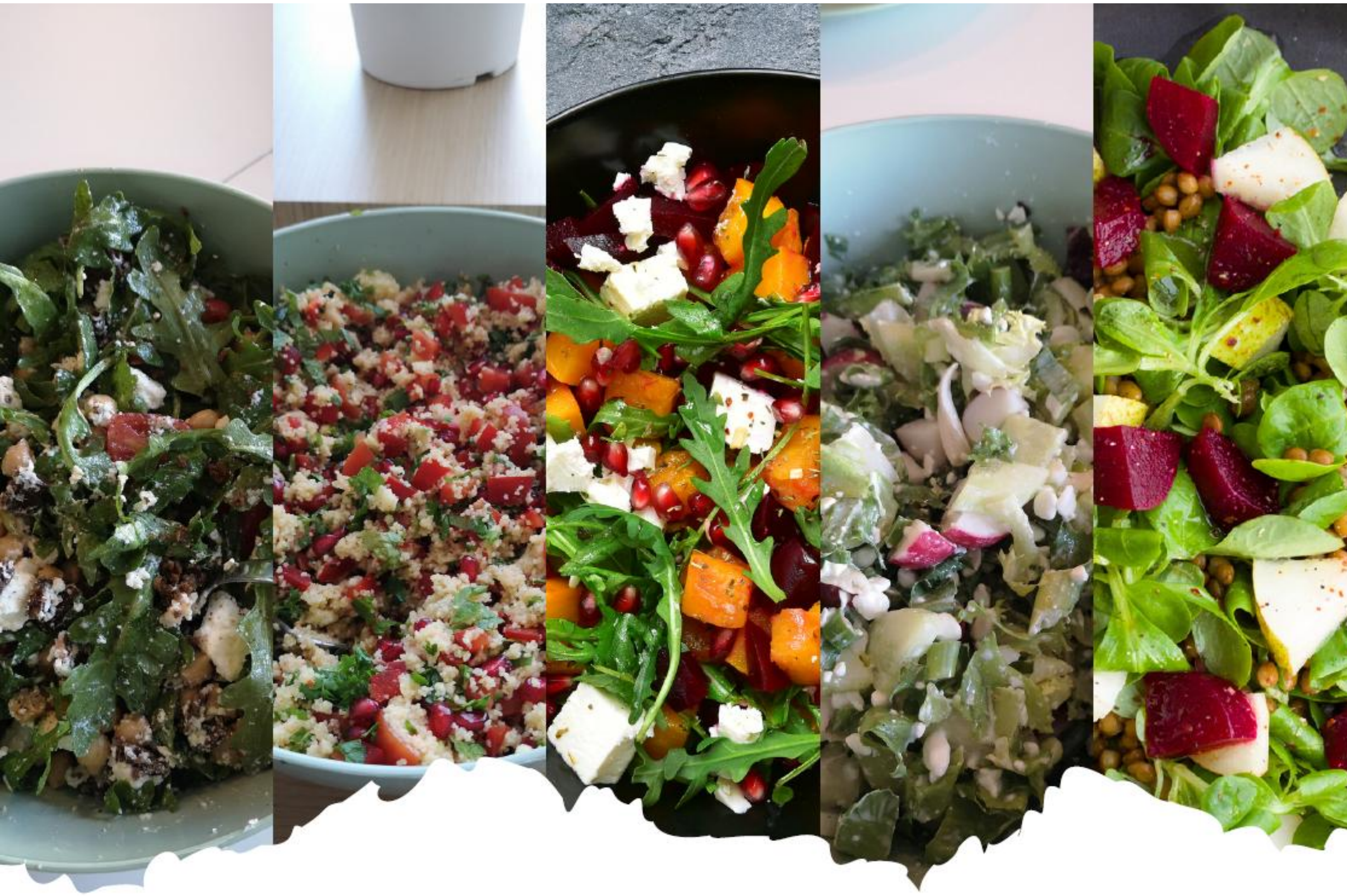

Healthy Recipes for Work

Take a moment to revisit the salad and meal ideas from our workshops — and make them part of your daily routine!

Remember, you can always adapt the ingredients to whatever you have in your fridge. Be creative, experiment, and enjoy every bite... to your health!

Prepared by: Aleksandra Hyży, MSc – Dietitian, Public Health Specialist

Medical University of Warsaw

1. Salad with cottage cheese

Portion size (standardized): ~260 g
Prep time: ~10 min
Serving suggestion: with bread (optional)

**Ingredients**	**Allergen flags**
- A pack of your favorite salad mix<br>- 1 long cucumber<br>- 2 bunches of radishes<br>- Half a bunch of chives<br>- 1 pack of cottage cheese<br>- Salt and pepper to taste<br>- Optional: your favorite herbs	• Contains: milk (cow's milk, lactose) — from cottage cheese.<br>• Gluten-free if served without bread.<br>**Fun fact**<br>Radish leaves are edible! You can add them to a salad or make pesto out of them. They have a slightly spicy, fresh flavor.

Nutrition values

	Energy [kcal]	Fat [g]	Saturated fat [g]	Carbohydrates [g]	Sugars [g]	Fibre [g]	Protein [g]	Salt [g]
Per batch (775g)	309.4	10.1 g	5.3 g	28 g	19 g	9.1 g	32.1 g	2.2 g
Per portion (~260g)	274	3.3	1.8	9.3	6.3	3	10.6	0.7

How to make it:
Wash all vegetables thoroughly – yes, the salad mix too (!) – and drain well. Cut the cucumber and radishes into smaller pieces. You can peel the cucumber or leave the skin – it's up to you. Chop the chives finely. Mix all vegetables in a bowl and add the cottage cheese along with the 'cream' – this will be the dressing. Season with salt, pepper, and herbs if you wish. Serve with your favorite bread.

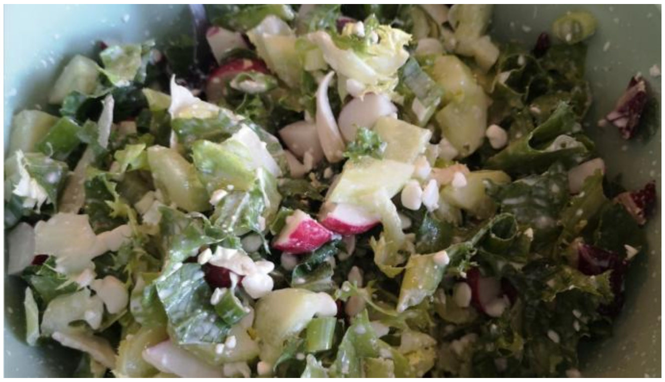

Data sources for nutrient values. Primary: Tabela składu i wartości odżywczej żywności (IŻŻ/NIZP-PZH, wyd. 2020). For "serek wiejski" values, typical Polish label averages for plain cottage cheese were used (~103 kcal, 5 g fat, 3.2 g SFA, 11 g protein, 0.6 g salt per 100 g). Warzywa świeże (sałaty, ogórek, rzodkiewka, szczypiorek) wg pozycji tabel IŻŻ 2020.

2. Vegetable sticks with yogurt dip

Portion size (standardized): ~220 g
Prep time: ~10 min
Serving suggestion: with bread (optional)

**Ingredients**	**Allergen flags**
- Carrots<br>- Celery stalks<br>- 1 cup of cottage cheese or natural yogurt<br>- Salt, pepper to taste<br>- Favorite herbs (e.g. chives, parsley)	• Contains: milk (cow's milk, lactose) — from cottage cheese.<br>• Gluten-free if served without bread.<br>**Tip**<br>You can also dip cucumber sticks, cherry tomatoes, roasted potatoes, sweet potatoes, and anything that keeps its shape after dipping in the sauce.

Nutrition values

	Energy [kcal]	Fat [g]	Saturated fat [g]	Carbohydrates [g]	Sugars [g]	Fibre [g]	Protein [g]	Salt [g]
Per batch (~857g)	359.5	9.9	5.4	44.8	27.9	16.9	31.5	3.1
Per portion (~220g)	91.8	2.5	1.4	11.5	7.1	4.4	8.1	0.8

How to make it:
Prepare the dip: mix cottage cheese or yogurt with the spices. Prepare the vegetables: wash, peel, remove any tough parts (like celery fibers), and cut into smaller pieces – sticks. Dip the vegetables in the yogurt or cottage cheese dip. Serve with your favorite bread.

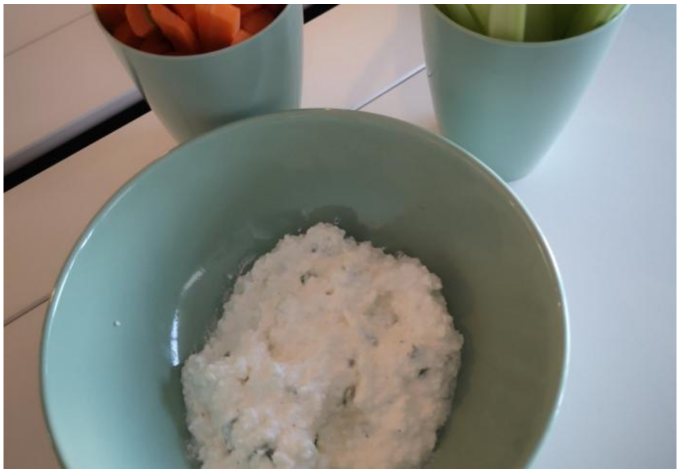

Primary source: Polish Food Composition Tables (*Tabela składu i wartości odżywczej żywności*, National Institute of Public Health – NIZP-PZH, 2020 edition)

3. Arugula, chickpea, and feta salad

Standard portion size: ~230g
Preparation time: ~10–12 min
Serving suggestion: serve as a light lunch or side dish

**Ingredients**	**Allergen information**
- 1 pack of arugula (150g)<br>- 1 pack of feta or salad cheese<br>- 1 can of chickpeas (240g drained)<br>- 150g cherry tomatoes<br>- 2–3 slices of pumpernickel bread<br>- A handful of pumpkin seeds<br>- Olive oil<br>- Salt, pepper<br>- Favorite herbs	• Contains: milk (from feta or salad cheese), gluten (from pumpernickel bread).<br>• May contain traces of sesame or nuts depending on the brand of pumpkin seeds.<br>**Fun fact**<br>You don't have to throw away the water from chickpeas – it's vegan protein! You can whip it with sugar to make vegan meringue.

Don't be afraid to experiment in the kitchen! Originally, this salad was supposed to have croutons, but I couldn't find them in the shop. Instead, I used pumpernickel – it works great here and has more fiber.

Nutrition values

	Energy [kcal]	Fat [g]	Saturated fat [g]	Carbohydrates [g]	Sugars [g]	Fibre [g]	Protein [g]	Salt [g]
Per batch (~915g)	1920.3	68.9	25.5	239.0	184.5	54.4	103.6	8.6
Per portion (~230g)	480.1	17.2	6.4	59.7	46.1	13.6	25.9	2.1

How to make it:

Wash the vegetables. Put the arugula in a bowl and cut the tomatoes into smaller pieces. Add them along with the drained chickpeas. Cut the cheese and pumpernickel into cubes and add to the bowl. Sprinkle with pumpkin seeds and mix. Season with olive oil, salt (careful – the cheese is salty), pepper, and herbs. Mix well and enjoy!

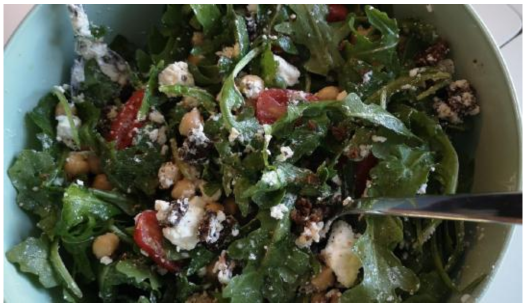

Primary source: Polish Food Composition Tables (*Tabela składu i wartości odżywczej żywności*, National Institute of Public Health – NIZP-PZH, 2020 edition)

4. Couscous salad with vegetables and pomegranate

Standard portion size: ~250 g
Preparation time: ~15 min
Serving suggestion: serve chilled or at room temperature

**Ingredients**	**Allergen information**
- 100g couscous<br>- 1 large red pepper<br>- 2 large bunches of parsley or coriander<br>- 150g cherry tomatoes<br>- Seeds from one small pomegranate<br>- Lemon juice<br>- Olive oil<br>- Salt, pepper<br>- Broth for couscous	• Contains: gluten (from couscous).<br>• May contain traces of sesame or nuts (depending on pomegranate processing).<br>**Fun fact**<br>Couscous is closer to pasta than to groats because of the production process. It's very easy to prepare – just pour hot liquid over it. You can serve it both with savory and sweet dishes.

Nutrition values

	Energy [kcal]	Fat [g]	Saturated fat [g]	Carbohydrates [g]	Sugars [g]	Fibre [g]	Protein [g]	Salt [g]
Per batch (~1006g)	765.5	21.4	3.2	130.8	111.0	19.8	22.5	0.4
Per portion ~250 g	191.5	5.3	0.8	32.7	27.8	5.0	5.6	0.1

How to make it:

Pour couscous into a bowl and pour hot broth over it so the liquid covers it by about 1 cm. Set aside to 'swell' and cool. Wash and dry the vegetables. Dice the pepper and tomatoes finely, and chop the parsley. Remove pomegranate seeds and add them to the bowl. Season the salad with plenty of lemon juice, olive oil, salt, pepper, and herbs. Enjoy!

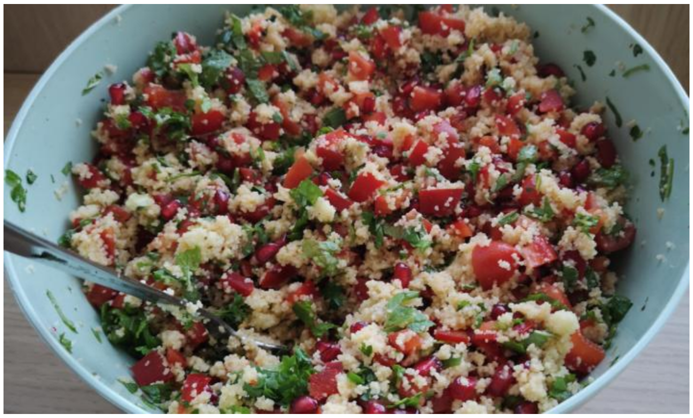

Primary source: Polish Food Composition Tables (*Tabela składu i wartości odżywczej żywności*, National Institute of Public Health – NIZP-PZH, 2020 edition)

5. Carrot cream soup with orange and ginger

Standard portion size: ~325 g
Preparation time: ~30 min
Serving suggestion: serve hot, topped with yogurt and roasted pumpkin seeds

Ingredients	**Allergen information**
- 10 carrots (600g)<br>- 1 parsley root (80g)<br>- 1 onion (120g)<br>- 2 cm piece of ginger (10g)<br>- 2 garlic cloves<br>- 3 cups unsalted vegetable broth<br>- Grated zest and juice of ½ orange<br>- 2 tablespoons olive oil or butter<br>- 3 tablespoons natural yogurt<br>- 1 tablespoon pumpkin seeds	• Contains: milk (from natural yogurt; lactose).<br>• May contain traces of nuts or sesame (from pumpkin seeds).<br>• Gluten-free when served without bread or other gluten-containing sides.

Nutrition values

	Energy [kcal]	Fat [g]	Saturated fat [g]	Carbohydrates [g]	Sugars [g]	Fibre [g]	Protein [g]	Salt [g]
Per batch (~1587g)	620.3	26.8	4.7	94.2	60.3	33.9	18.5	2.0
Per portion (~325 g)	127.1	5.3	1.0	19.3	12.6	6.8	4.0	0.4

How to make it:
Peel carrots, parsley root, onion, ginger, and garlic. Cut the carrots, parsley, and onion into cubes. Roast the ginger over a gas flame or in an oven preheated to 150°C. Add the parsley and carrots to a pot with broth and cook until tender. On heated olive oil or butter, sauté onion and garlic until translucent, then add ginger, stew for 3 minutes, and transfer to the broth. Add orange zest and juice, cook for 5 minutes. Blend the soup until smooth. For a perfectly creamy texture, you can strain it through a sieve. Just before serving, add natural yogurt and mix thoroughly. Serve in bowls and sprinkle with roasted pumpkin seeds.

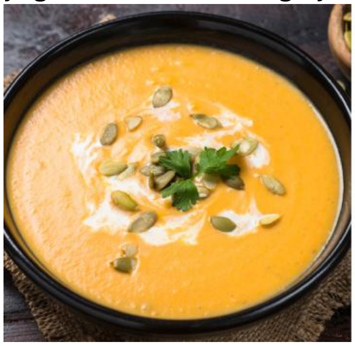
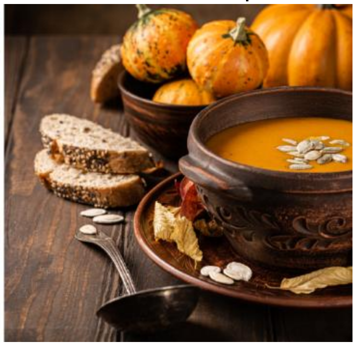

Primary source: Polish Food Composition Tables (*Tabela składu i wartości odżywczej żywności*, National Institute of Public Health – NIZP-PZH, 2020 edition)

6. Roasted eggplant and pepper cream soup

Standard portion size: ~400 g
Preparation time: ~35–40 min
Serving suggestion: serve hot with fresh herbs or a slice of whole-grain bread

**Ingredients**	**Allergen information**
- 1 kg eggplants<br>- 4 red peppers<br>- 2 tablespoons olive oil<br>- 2 garlic cloves<br>- 4 tomatoes<br>- 750 ml broth<br>- Sea salt and freshly ground pepper	• Contains: none of the major allergens.<br>• Suitable for: vegans and vegetarians.<br>• Gluten-free.

Nutrition values

	Energy [kcal]	Fat [g]	Saturated fat [g]	Carbohydrates [g]	Sugars [g]	Fibre [g]	Protein [g]	Salt [g]
Per batch (~3630g)	982.7	30.5	4.6	181.8	118.3	63.5	36.7	2.7
Per portion (~400 g)	110.8	3.6	0.5	20.1	13.1	7.0	4.1	0.3

How to make it:

Preheat the oven to 190°C. Wash the vegetables, cut the eggplants in half, and peppers into quarters, removing seeds. Place eggplants and peppers skin side up on a baking sheet and roast for 10 minutes. After roasting, peel off the skins and cut into large cubes. Blanch tomatoes in boiling water, peel, and chop. In a wok or a large pan, heat the olive oil and fry the sliced garlic. Then add the tomatoes and simmer, stirring often. After 2 minutes, add the eggplants and peppers, pour in the broth, and simmer for 4–5 minutes, stirring occasionally. Season with salt and pepper, stir again, and blend the soup until smooth. Serve hot.

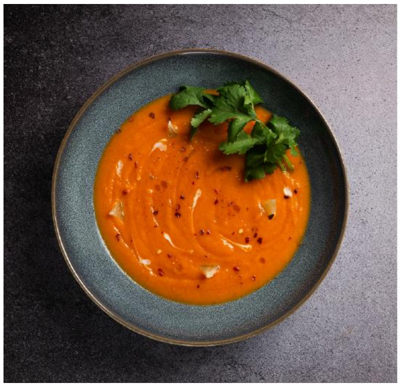
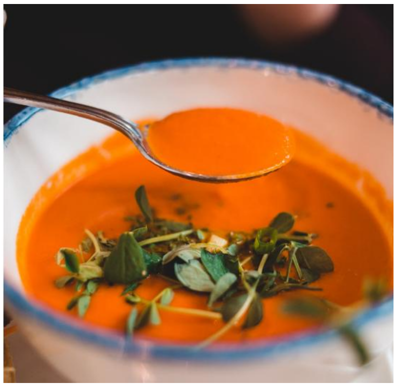

Primary source: Polish Food Composition Tables (*Tabela składu i wartości odżywczej żywności*, National Institute of Public Health – NIZP-PZH, 2020 edition)
