# Supplementary material for: A Brief Participatory Workplace Intervention on Dietary Barriers and Healthy Eating Intentions Among Employees: A Pilot Study
Source: Nutrients. 2025 Oct 27;17(21):3371. doi: 10.3390/nu17213371 (PMC12610673; doi:10.3390/nu17213371)
Supplement: Supplementary file 1 [file nutrients-17-03371-s001.zip › Supplementary S1.pdf]

Healthy meals at work

Knowledge compendium after the workshop

Author: Aleksandra Hyży, Medical University of Warsaw

Purpose of the material

The aim of this material is to provide k nowledge and practical tips on healthy eating at work. After the workshop, you already know:

- What a healthy work meal is
- How to compose a second breakfast or lunch
- What realistically hinders the introduction of healthy habits
- Examples of good practices and solutions used around the world

What is a healthy meal at work?

A meal that:
• Provides energy and supports concentration
• Prevents sudden hunger and snacking on sweets
• Keeps blood sugar levels stable
• Is filling but doesn't cause heaviness

Examples:

• Whole-grain sandwiches with vegetables and lean protein (e.g. egg, hummus, cottage cheese)
• Natural yogurt with fruit and nuts
• Oatmeal with plant-based milk and toppings (fruit, seeds, cinnamon)
• Vegetables with hummus and a slice of bread

Principles of healthy eating – what's new?

Modern approaches to healthy nutrition are based on simple, scientifically proven rules.

Macronutrients (proportion of daily energy):

• Carbohydrates (C) – 50% of energy: whole-grain products, vegetables, fruits
• Fats (F) – 30–35% of energy: mainly plant fats (rapeseed oil, olive oil, nuts, avocado)
• Protein (P) – 10–15% of energy: lean meat, fish, legumes, eggs, dairy

Information for you: 1 g protein = 4 kcal; 1 g carbohydrates = 4 kcal; 1 g fat = 9 kcal

What prevents us from eating healthy?

- Lack of time to prepare and eat
- No refrigerator or microwave at work
- Poor availability of healthy options nearby
- Habits and the desire to 'reward yourself' with food
- Social pressure ('everyone eats fast food')

Exercise: lunchbox building blocks

Compose your meal from the ingredients below:

- Protein source: egg, tofu, fish, chicken breast, beans
- Carbohydrate source: groats, rice, whole-grain bread, pasta
- Vegetables: broccoli, arugula, tomato, carrot
- Healthy fats: pumpkin seeds, olive oil, avocado
- Flavor add-ons: herbs, spices, lemon
- Something sweet: fruit, chocolate, honey
- Water to drink

Your ideas:
1...
2...
3...
4...
5...
6...
7...
8...
9...
10...

Think and answer the questions:

Where do you get your lunch for work?
....
....
....
What does a 'healthy lunch' mean to you?
....
....
....

What can you and do you like to eat at work?

....
....
....

What's going on elsewhere? – practices from other countries

- Lectures and workshops with dietitians
- Individual consultations
- Employee health promotion programs
- Free fruits and vegetables
- Subsidized meals with high nutritional value
- Labels in canteens informing about the nutritional value of dishes

What's Next?

To make changes lasting:

- Plan your meals – shopping list, cooking for several days
- Talk with your team about joint healthy initiatives
- Make room for healthy choices – in the kitchen, fridge, canteen

Thank you!

Your change can start today – even with one healthy sandwich or oatmeal. Support yourself and your work environment in building healthy habits.
