# Supplementary figures and images for: A Brief Participatory Workplace Intervention on Dietary Barriers and Healthy Eating Intentions Among Employees: A Pilot Study
Source: Nutrients. 2025 Oct 27;17(21):3371. doi: 10.3390/nu17213371 (PMC12610673; doi:10.3390/nu17213371)

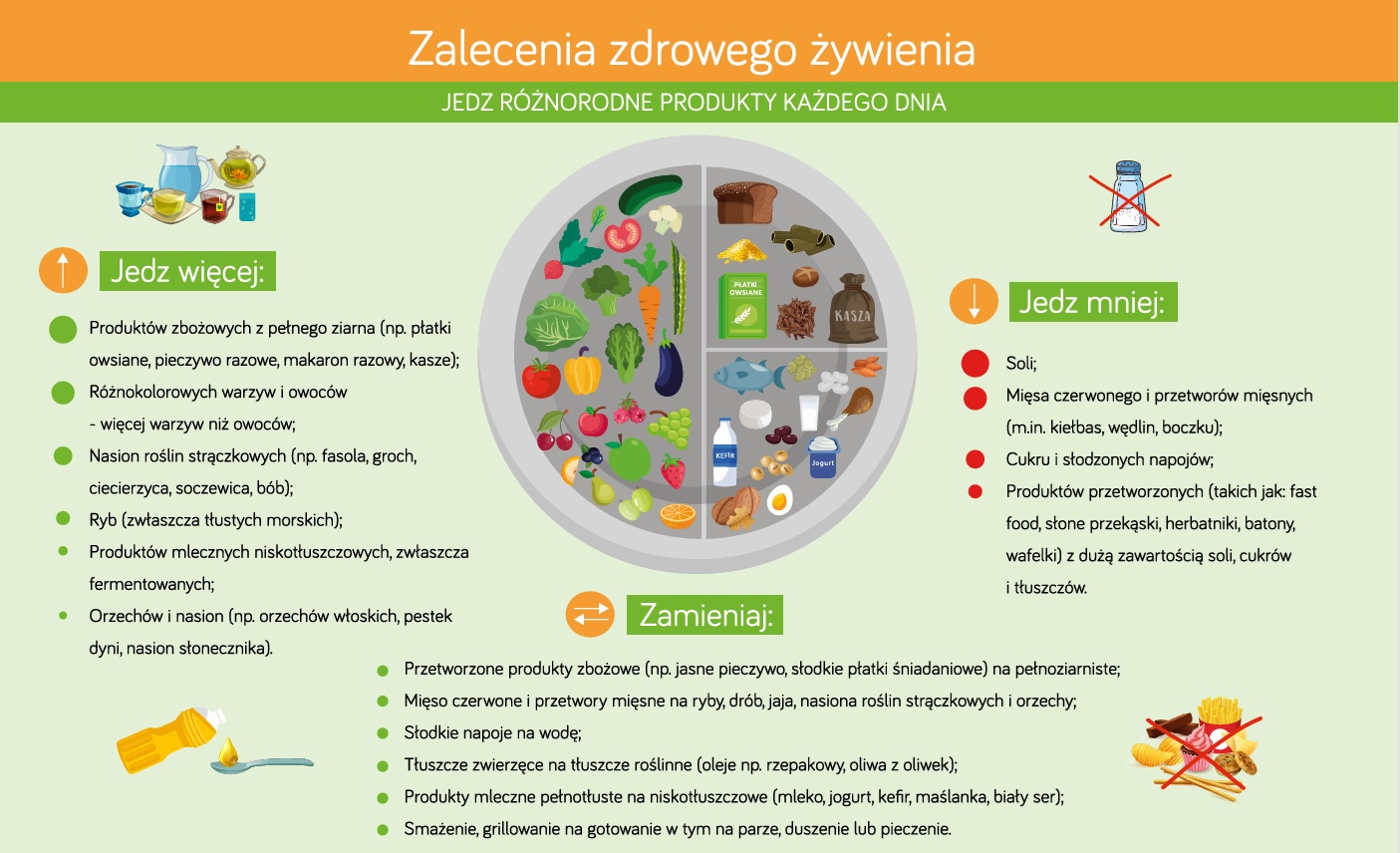

Supplement: Supplementary file 1 [file nutrients-17-03371-s001.zip › Supplementary S2.jpg]
